# TAO-DFT investigation of electronic properties of linear and cyclic carbon chains

**DOI:** 10.1038/s41598-020-70023-z

**Published:** 2020-08-04

**Authors:** Sonai Seenithurai, Jeng-Da Chai

**Affiliations:** 1 Department of Physics, National Taiwan University, Taipei, 10617 Taiwan; 2 Center for Theoretical Physics and Center for Quantum Science and Engineering, National Taiwan University, Taipei, 10617 Taiwan

**Keywords:** Theoretical chemistry, Computational chemistry, Density functional theory, Quantum chemistry, Chemical physics, Nanoscale materials, Nanowires, Chemistry, Nanoscience and technology, Materials science, Theory and computation, Electronic structure

## Abstract

It has been challenging to adequately investigate the properties of nanosystems with radical nature using conventional electronic structure methods. We address this challenge by calculating the electronic properties of linear carbon chains (*l*-CC[*n*]) and cyclic carbon chains (*c*-CC[*n*]) with *n* = 10–100 carbon atoms, using thermally-assisted-occupation density functional theory (TAO-DFT). For all the cases investigated, *l*-CC[*n*]/*c*-CC[*n*] are ground-state singlets, and *c*-CC[*n*] are energetically more stable than *l*-CC[*n*]. The electronic properties of *l*-CC[*n*]/*c*-CC[*n*] reveal certain oscillation patterns for smaller *n*, followed by monotonic changes for larger *n*. For the smaller carbon chains, odd-numbered *l*-CC[*n*] are more stable than the adjacent even-numbered ones; *c*-CC[$4m+2$]/*c*-CC[4*m*] are more/less stable than the adjacent odd-numbered ones, where *m* are positive integers. As *n* increases, *l*-CC[*n*]/*c*-CC[*n*] possess increasing polyradical nature in their ground states, where the active orbitals are delocalized over the entire length of *l*-CC[*n*] or the whole circumference of *c*-CC[*n*].

## Introduction

Carbon is the most versatile element in forming various structures. In bulk phase, graphite and diamond, which are well-known materials, have been used for centuries. In nanoforms, fullerenes and graphene have been studied in detail for decades. In general, nanostructures can be classified into three categories: zero-dimensional (0D), one-dimensional (1D), and two-dimensional (2D) nanomaterials. Carbon forms all these nanostructures with unique shapes and properties. Over the past few decades, carbon nanomaterials have been widely studied, and applied in diverse industries^1–3^.

A number of carbon nanostructures have been synthesized and applied in different fields. The 0D carbon nanomaterials include clusters, quantum dots, nanoflakes, and buckyballs^3^. Among them, the C$_{60}$ fullerene molecule (containing 12 pentagons and 20 hexagons), where the carbon atoms are $sp^{2}$–$sp^{3}$-hybridized, has been a popular carbon nanomaterial^1^. The discovery of C$_{60}$ has led to the flourishment of carbon nanomaterials in various ways.

Graphite is a bulk layered material, where the $sp^{2}$-hybridized carbon atoms in each layer are arranged in a hexagonal lattice. The 2D carbon nanomaterial, graphene, can be obtained by mechanically exfoliating a single layer of carbon atoms from graphite^2^. Thus, graphene, which is a perfect arrangement of hexagons made up of $sp^{2}$-hybridized carbon atoms in a 2D planar surface, can be the thinnest (i.e., single-atom-thick) material synthesized ever. Graphene is a zero-gap semiconductor or semimetal with massless Dirac fermions with linear dispersion at low energy. Because of the Dirac-cone feature, graphene has huge potential in electronics applications^2^. The discovery of graphene has also led to the discovery of other 2D materials. Besides, if a graphene sheet can be rolled up to form a seamless cylinder, one obtains a carbon nanotube (CNT), which belongs to the class of 1D nanostructures. Note that CNTs were first observed by Iijima in 1991^4^, well before the separation and characterization of graphene. On the basis of the direction of rolling (chirality), CNTs can be classified into three groups: chiral, zigzag, and armchair CNTs, which can have rather different electronic properties.

**Figure 1:**
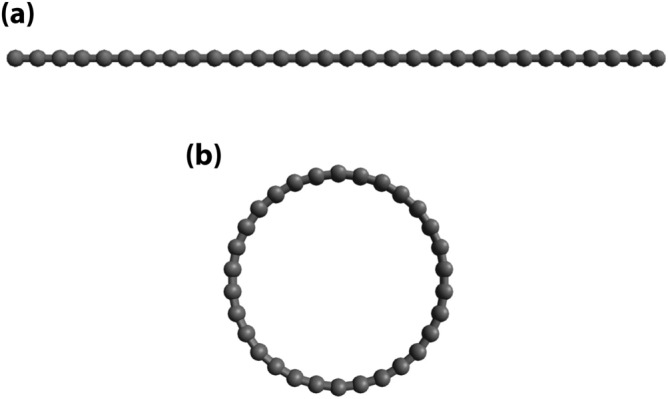
Structures of (**a**) *l*-CC[30] and (**b**) *c*-CC[30], each consisting of 30 carbon atoms.

Apart from these well studied carbon nanoallotropes, linear carbon chains (i.e., also belonging to the class of 1D nanostructures), where the carbon atoms are *sp*-hybridized, have recently gained much attention because of their interesting physical and chemical properties^5–18^. A linear carbon chain consisting of *n* carbon atoms (for brevity, denoted as *l*-CC[*n*] (see Fig. 1a)) is an ideal 1D carbon nanomaterial. Kroto et al. originally designed an experiment for explaining the formation mechanism of carbon chains in outer space, which led to the serendipitous discovery of C$_{60}$^1^. In attempting to synthesize *l*-CC[*n*] (i.e., the holy grail of truly 1D carbon allotropes), significant progresses have been made recently^6,11,14^. Owing to their high reactivity, pristine linear carbon chains have not yet been reported. However, the linear carbon chains (containing up to 100 carbon atoms) supported inside multi-walled CNTs were realized in 2003 by Zhao et al.^6^. In 2015, Andrade et al.^11^ observed the carbon chains inside CNTs under high pressures. In 2016, Shi et al.^14^ demonstrated the large-scale syntheses of linear carbon chains inside double-walled CNTs by using the confined space inside the tube as a nanoreactor to grow ultra-long carbon chains (containing up to 6400 carbon atoms) in large quantities^14,15^. Linear carbon chains can be potential candidates for nanodevices, molecular electronics, and the building blocks of novel hybrid nanomaterials (e.g., *sp*–$sp^{2}$ and *sp*–$sp^{3}$ hybridized materials) by integrating with other nanostructures^8,9,17^. On the theoretical side, a number of relevant calculations are available^9^. The calculations showed that the linear carbon chains inside single-walled CNTs can modify the electronic properties of pristine single-walled CNTs significantly. It has been found that the chirality of the enclosing nanotubes can affect the properties of linear carbon chains^18^. Besides, recent theoretical studies showed that linear carbon chains have excellent mechanical and electrical properties^9,10,14^.

On the other hand, cyclic carbon chains (for brevity, denoted as *c*-CC[*n*] (see Fig. 1b)), which are the monocyclic isomers of linear carbon chains, have also attracted considerable attention in recent years^19–38^. Note that *c*-CC[*n*] (where the carbon atoms are also *sp*-hybridized) are hypothesized to be the building blocks of fullerenes in the initial stages of growth^21^. Interestingly, *c*-CC[$4m+2$] (where *m* are positive integers) have been found to possess high stability^24,25^. Among them, *c*-CC[18] has recently been synthesized^26^, and some of the electronic properties of *c*-CC[18] have been reported. There are also a few theoretical studies on the electronic properties and applications of *c*-CC[18] and other cyclic carbon chains^22,27–38^. For example, *c*-CC[18] has been found to possess electron-acceptor properties, and can be dubbed as the smallest all-carbon electron acceptor^28^.

In general, it remains extremely difficult to synthesize both *l*-CC[*n*] and *c*-CC[*n*]. Accordingly, theoretical studies are complementary, and may provide immense information on the properties of *l*-CC[*n*] and *c*-CC[*n*]. In a recent theoretical study, it has been reported that very small *l*-CC[*n*] (*n* = 5–10) with even *n* values possess diradical nature in their ground states^16^. Therefore, it can be anticipated that the larger *l*-CC[*n*] and *c*-CC[*n*] could also possess radical character due to the low dimensionality of *l*-CC[*n*] and *c*-CC[*n*], respectively^39^. Despite its success in describing some ground-state properties, Kohn-Sham density functional theory (KS-DFT)^40^ can yield unreliable results for systems with radical nature^41^, when the conventional XC density functionals are employed. Typically, multi-reference (MR) electronic structure methods, such as the complete-active-space self-consistent-field (CASSCF), complete-active-space second-order perturbation theory (CASPT2), and related methods^42–48^, are required to reliably predict the energy and related properties of systems with radical nature. Nonetheless, accurate MR electronic structure methods can be prohibitively expensive for large systems, and hence may not be practical for studying the properties of the larger *l*-CC[*n*] and *c*-CC[*n*].

Recently, thermally-assisted-occupation density functional theory (TAO-DFT)^49^, which is a density functional theory with fractional orbital occupation numbers, has been formulated to tackle such challenging problems (i.e., nanosystems with radical nature), wherein an entropy contribution term (i.e., a function of the fictitious temperature $\theta$ and orbital occupation numbers) can approximately describe strong static correlation even when the simplest local density approximation (LDA) XC density functional is employed. In TAO-DFT, one can also adopt more sophisticated XC density functionals, such as semilocal^50^, global hybrid^51^, and range-separated hybrid^51,52^ XC density functionals. Besides, aiming to improve the accuracy of TAO-DFT for a wide range of applications, a self-consistent scheme determining the fictitious temperature $\theta$ in TAO-DFT has been recently proposed^53^. Since TAO-DFT is a computationally efficient electronic structure method, a number of strongly correlated electron systems at the nanoscale have been studied using TAO-DFT in recent years^16,54–62^. Besides, TAO-DFT has been recently shown to be useful in describing the vibrational spectra of molecules with radical nature^63^. In addition, TAO-DFT and related methods have recently been employed to investigate the electronic properties of several nanosystems with radical nature^64,65^, and have also been combined with the linear-scaling divide-and-conquer approach for the study of large systems with strong static correlation effects^66^.

Therefore, in the present study, we carry out TAO-DFT calculations to investigate the electronic properties of *l*-CC[*n*]/*c*-CC[*n*] with *n* = 10–100. Specifically, we report the electronic properties, such as the singlet-triplet energy gap, singlet-quintet energy gap, vertical ionization potential, vertical electron affinity, fundamental gap, symmetrized von Neumann entropy, active orbital occupation numbers, real-space representation of active orbitals, and relative stability of *l*-CC[*n*]/*c*-CC[*n*]. For considerably large values of *n*, we show that both *l*-CC[*n*] and *c*-CC[*n*] are polyradicals in their ground states, playing an important role in determining their electronic properties.

## Computational details

All geometry optimizations and other calculations are performed with Q-Chem 4.4^67^, using the 6-31G(d) basis set (i.e., a valence double-zeta polarized basis set) with a numerical quadrature that consists of 75 points in the Euler-Maclaurin radial grid and 302 points in the Lebedev angular grid. We carry out calculations using TAO-LDA^49^, which is TAO-DFT employing the LDA XC and $\theta$-dependent density functionals, with the recommended fictitious temperature $\theta$ = 7 mhartree^49^.

In several recent studies^46,48,49,54,55^, the orbital occupation numbers obtained from TAO-LDA (with $\theta$ = 7 mhartree) have been found to be qualitatively similar to the natural orbital occupation numbers obtained from the variational two-electron reduced-density-matrix-driven CASSCF (v2RDM-CASSCF) method (i.e., an accurate MR electronic structure method), leading to a similar trend for the radical nature of several systems.

## Results and discussion

### Singlet-triplet energy gap and singlet-quintet energy gap

**Figure 2:**
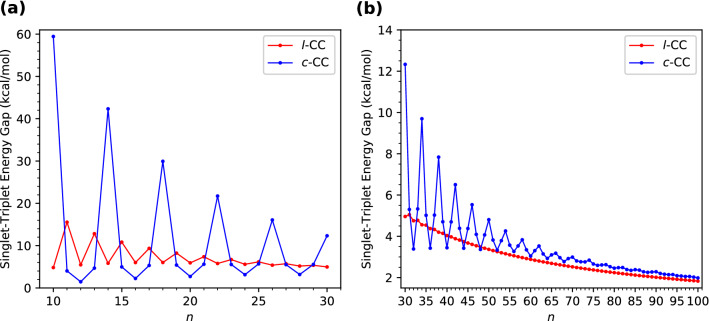
Singlet-triplet energy gap of *l*-CC[*n*]/*c*-CC[*n*] with (**a**) *n* = 10–30 and (**b**) *n* = 30–100, calculated using spin-unrestricted TAO-LDA.

**Figure 3:**
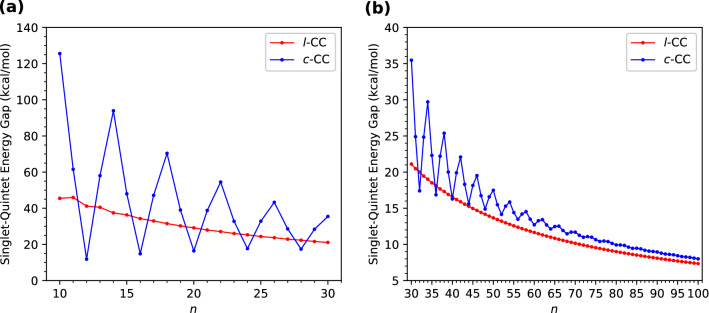
Singlet-quintet energy gap of *l*-CC[*n*]/*c*-CC[*n*] with (**a**) *n* = 10–30 and (**b**) *n* = 30–100, calculated using spin-unrestricted TAO-LDA.

Aiming to obtain the energetically preferred spin state (i.e., the ground state) of *l*-CC[*n*]/*c*-CC[*n*], we obtain the energies of *l*-CC[*n*]/*c*-CC[*n*] for the lowest singlet, triplet, and quintet states by optimizing the corresponding structures with spin-unrestricted TAO-LDA, and thereafter compute the singlet-triplet energy gap of *l*-CC[*n*]/*c*-CC[*n*] using

1 $$\begin{aligned} E_{\text {ST}} = E_{\text {T}} - E_{\text {S}}, \end{aligned}$$

and compute the singlet-quintet energy gap of *l*-CC[*n*]/*c*-CC[*n*] using

2 $$\begin{aligned} E_{\text {SQ}} = E_{\text {Q}} - E_{\text {S}}, \end{aligned}$$

where $E_{\text {Q}}$, $E_{\text {T}}$, and $E_{\text {S}}$ are the lowest quintet, triplet, and singlet energies, respectively, of *l*-CC[*n*]/*c*-CC[*n*] (also see Section I, Figure S1, and Figure S2 in Supplementary Information (SI)). According to their definitions, the $E_{\text {ST}}$ and $E_{\text {SQ}}$ reported in this work are the adiabatic singlet-triplet energy gap and adiabatic singlet-quintet energy gap, respectively, of *l*-CC[*n*]/*c*-CC[*n*]. The $E_{\text {ST}}$ and $E_{\text {SQ}}$ values of *l*-CC[*n*]/*c*-CC[*n*] as functions of the number of carbon atoms are presented in Figs. 2 and 3, respectively (also see Tables S1 and S2 in SI).

Since the $E_{\text {ST}}$ and $E_{\text {SQ}}$ values remain positive, the *l*-CC[*n*]/*c*-CC[*n*] studied are all ground-state singlets. The $E_{\text {ST}}$ values of the smaller *l*-CC[*n*] exhibit an odd-even oscillation pattern in which the $E_{\text {ST}}$ values of odd-numbered *l*-CC[*n*] are larger than those of the adjacent even-numbered ones. However, such oscillations decrease with increasing *n*, and eventually disappear for considerably large *n*. Accordingly, the $E_{\text {ST}}$ values of *l*-CC[*n*] are oscillatory only for smaller values of *n* (up to *n* = 33), and become monotonically decreasing for larger *n*. The $E_{\text {SQ}}$ values of *l*-CC[*n*] decrease essentially monotonically with *n* (i.e., except only for *n* = 10).

By contrast, the $E_{\text {ST}}$ and $E_{\text {SQ}}$ values of the smaller *c*-CC[*n*] display distinct oscillation patterns in which the $E_{\text {ST}}$ and $E_{\text {SQ}}$ values of *c*-CC[$4m+2$]/*c*-CC[4*m*] are larger/smaller than the $E_{\text {ST}}$ and $E_{\text {SQ}}$ values, respectively, of the adjacent odd-numbered ones, where *m* are positive integers. Nevertheless, with the increase of molecular size, these oscillations are progressively reduced, and eventually absent for sufficiently large *n*. Therefore, the $E_{\text {ST}}$ and $E_{\text {SQ}}$ values of *c*-CC[*n*] are oscillatory only for smaller *n* (up to *n* = 94 for $E_{\text {ST}}$ and up to *n* = 85 for $E_{\text {SQ}}$), and become monotonically decreasing for larger values of *n*. Note that the reasons for these oscillation patterns have been recently provided in a quantum Monte Carlo study^34^.

Understanding the $E_{\text {ST}}$ values is essential for applications that involve harnessing energy through the singlet-fission phenomenon^68^. Consequently, the $E_{\text {ST}}$ values of *l*-CC[*n*]/*c*-CC[*n*] reported in the present study can provide insight into the singlet-fission phenomenon. For other relevant applications, it is worth mentioning that the electronic transport properties of *c*-CC[18]-based molecular devices have been recently studied^38^.

### Vertical ionization potential, vertical electron affinity, and fundamental gap

**Figure 4:**
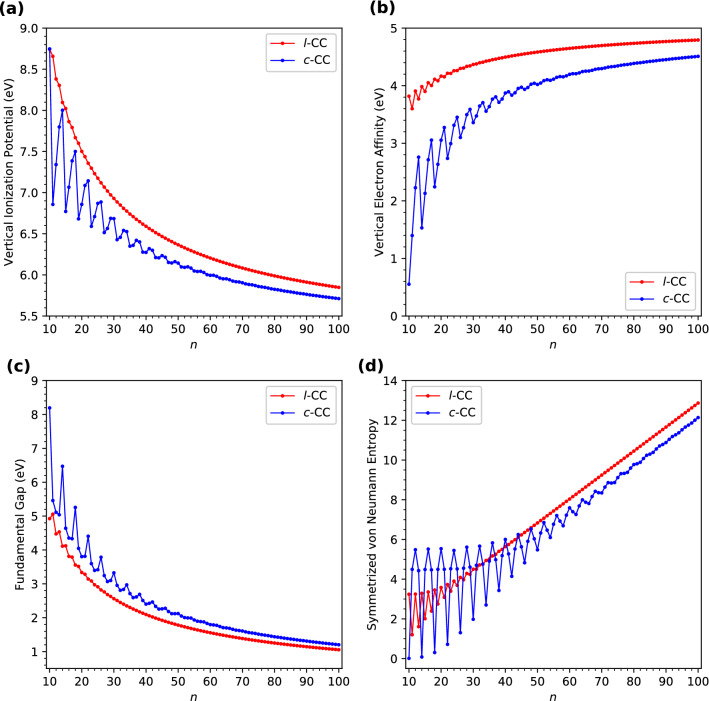
(**a**) Vertical ionization potential, (**b**) vertical electron affinity, (**c**) fundamental gap, and (**d**) symmetrized von Neumann entropy of ground-state *l*-CC[*n*]/*c*-CC[*n*] with *n* = 10–100, calculated using spin-unrestricted TAO-LDA.

Here, we assess if *l*-CC[*n*]/*c*-CC[*n*] are promising for photovoltaic applications. For a molecule in its ground state, the vertical ionization potential ($\text {IP}_{v}$) is the energy required to remove an electron from the molecule without affecting the molecular geometry, the vertical electron affinity ($\text {EA}_{v}$) is the energy released when an electron is added to the molecule without affecting the molecular geometry, and the fundamental gap ($E_{g}$) is the difference between $\text {IP}_{v}$ and $\text {EA}_{v}$ (i.e., $E_{g} = \text {IP}_{v} - \text {EA}_{v}$). Accordingly, in this work, we calculate the vertical ionization potential of ground-state *l*-CC[*n*]/*c*-CC[*n*] using

3 $$\begin{aligned} \text {IP}_{v} = {E}_{N-1} - {E}_{N}, \end{aligned}$$

the vertical electron affinity of ground-state *l*-CC[*n*]/*c*-CC[*n*] using

4 $$\begin{aligned} \text {EA}_{v} = {E}_{N} - {E}_{N+1}, \end{aligned}$$

and the fundamental gap of ground-state *l*-CC[*n*]/*c*-CC[*n*] using

5 $$\begin{aligned} E_{g} = {E}_{N+1} + {E}_{N-1} - 2 {E}_{N}, \end{aligned}$$

where ${E}_{N}$ is the total energy of the *N*-electron molecule (i.e., *l*-CC[*n*]/*c*-CC[*n*]) at the ground-state (i.e., lowest singlet state) geometry, obtained with spin-unrestricted TAO-LDA. The $\text {IP}_{v}$ (Fig. 4a), $\text {EA}_{v}$ (Fig. 4b), and $E_{g}$ (Fig. 4c) values of ground-state *l*-CC[*n*]/*c*-CC[*n*] are plotted as functions of *n* (also see Tables S3 and S4 in SI).

As *n* increases, the $\text {IP}_{v}$ value of *l*-CC[*n*] decreases monotonically, showing a very slight odd-even pattern only for very small *n*. The $\text {EA}_{v}$/$E_{g}$ values of the smaller *l*-CC[*n*] reveal an odd-even oscillation pattern in which the $\text {EA}_{v}$/$E_{g}$ values of odd-numbered *l*-CC[*n*] are smaller/larger than those of the adjacent even-numbered ones. Nevertheless, with the increase of *n*, these oscillations are progressively reduced, and ultimately absent for sufficiently large *n*. Accordingly, the $\text {EA}_{v}$/$E_{g}$ values of *l*-CC[*n*] are oscillatory only for smaller *n* (up to *n* = 21 for $\text {EA}_{v}$ and up to *n* = 15 for $E_{g}$), and become monotonically increasing/decreasing for larger values of *n*.

On the other hand, the $\text {IP}_{v}$/$\text {EA}_{v}$/$E_{g}$ values of the smaller *c*-CC[*n*] display a distinct oscillation pattern in which the $\text {IP}_{v}$/$\text {EA}_{v}$/$E_{g}$ values of *c*-CC[$4m+2$] are larger/smaller/larger than those of the adjacent odd-numbered ones, where *m* are positive integers. Nonetheless, such oscillations are gradually reduced, and eventually absent with the increase of molecular size. Therefore, the $\text {IP}_{v}$/$\text {EA}_{v}$/$E_{g}$ values of *c*-CC[*n*] are oscillatory only for smaller values of *n* (up to *n* = 61 for $\text {IP}_{v}$, up to *n* = 58 for $\text {EA}_{v}$, and up to *n* = 46 for $E_{g}$), and become monotonically decreasing/increasing/decreasing for larger *n*.

For each *n*, the $E_{g}$ value of *c*-CC[*n*] is larger than that of *l*-CC[*n*]. Besides, the $E_{g}$ values of *l*-CC[*n*] (with *n* = 24–100) and *c*-CC[*n*] (with *n* = 31–100) are in the range of 1 to 3 eV, showing promise for their applications in nanophotonics.

### Symmetrized von Neumann entropy

To assess the radical nature of ground-state (i.e., lowest singlet state) *l*-CC[*n*]/*c*-CC[*n*], we calculate the symmetrized von Neumann entropy^16,50,51,54,56–62,69^

6 $$\begin{aligned} S_{\text {vN}} = -\frac{1}{2} \sum _{\sigma =\uparrow ,\downarrow } \sum _{i=1}^{\infty } \bigg \lbrace f_{i,\sigma }\ \text {ln}(f_{i,\sigma }) + (1-f_{i,\sigma })\ \text {ln}(1-f_{i,\sigma }) \bigg \rbrace , \end{aligned}$$

using spin-unrestricted TAO-LDA. Here, the occupation number $f_{i,\sigma }$ of the $i$th $\sigma$-spin orbital (i.e., up-spin orbital or down-spin orbital) obtained with spin-unrestricted TAO-LDA, which ranges from 0 to 1, is closely related to the occupation number of the $i$th $\sigma$-spin natural orbital^49–51^. For a molecule with nonradical nature, the occupation numbers of all spin-orbitals should be close to either 0 or 1, leading to insignificant contributions to the corresponding $S_{\text {vN}}$ value. However, for a molecule with pronounced radical nature, the occupation numbers of active spin-orbitals (i.e., the spin-orbitals that have considerable fractional occupations) can be very different from 0 and 1 (e.g., between 0.1 and 0.9); hence, the corresponding $S_{\text {vN}}$ value is expected to increase when the occupation numbers of active spin-orbitals become closer to 0.5 and/or the number of active spin-orbitals increases.

As shown in Fig. 4d, the $S_{\text {vN}}$ values of ground-state *l*-CC[*n*] and *c*-CC[*n*] possess rather different oscillation patterns (also see Tables S3 and S4 in SI). The $S_{\text {vN}}$ values of the smaller *l*-CC[*n*] exhibit an odd-even oscillation pattern in which the $S_{\text {vN}}$ values of odd-numbered *l*-CC[*n*] are smaller than those of the adjacent even-numbered ones. Nonetheless, when the system size increases, such oscillations are gradually damped, and ultimately absent for considerably large *n*. Therefore, the $S_{\text {vN}}$ values of *l*-CC[*n*] are oscillatory only for smaller *n* (up to *n* = 29), and become monotonically increasing for larger *n*. By contrast, the $S_{\text {vN}}$ values of the smaller *c*-CC[*n*] reveal a distinct oscillation pattern in which the $S_{\text {vN}}$ values of *c*-CC[$4m+2$]/*c*-CC[4*m*] are smaller/larger than those of the adjacent odd-numbered ones, where *m* are positive integers. Nevertheless, with the increase of molecular size, these oscillations are progressively reduced, and eventually absent for sufficiently large *n*. Accordingly, the $S_{\text {vN}}$ values of *c*-CC[*n*] are oscillatory only for smaller *n* (up to *n* = 73), and become monotonically increasing for larger *n*. Since the $S_{\text {vN}}$ value can be regarded as a quantitative measure of the radical nature of a molecule, the larger *l*-CC[*n*]/*c*-CC[*n*] are expected to exhibit increasing polyradical nature in their ground states.

### Active orbital occupation numbers

To understand the reason that the symmetrized von Neumann entropy grows with the size of *l*-CC[*n*]/*c*-CC[*n*], it is instructive and informative to present the active orbital occupation numbers of ground-state *l*-CC[*n*]/*c*-CC[*n*] (consisting of *N* electrons), obtained with spin-restricted TAO-LDA, wherein the highest occupied molecular orbital (HOMO) is defined as the $(N/2)$th orbital, the lowest unoccupied molecular orbital (LUMO) is defined as the $(N/2+1)$th orbital, and so forth^49,51,58,60–62^. Here, the active orbitals are regarded as the orbitals with an occupation number ranging from 0.2 to 1.8 (i.e., the active spin-orbitals are regarded as the spin-orbitals with an occupation number ranging from 0.1 to 0.9).

**Figure 5:**
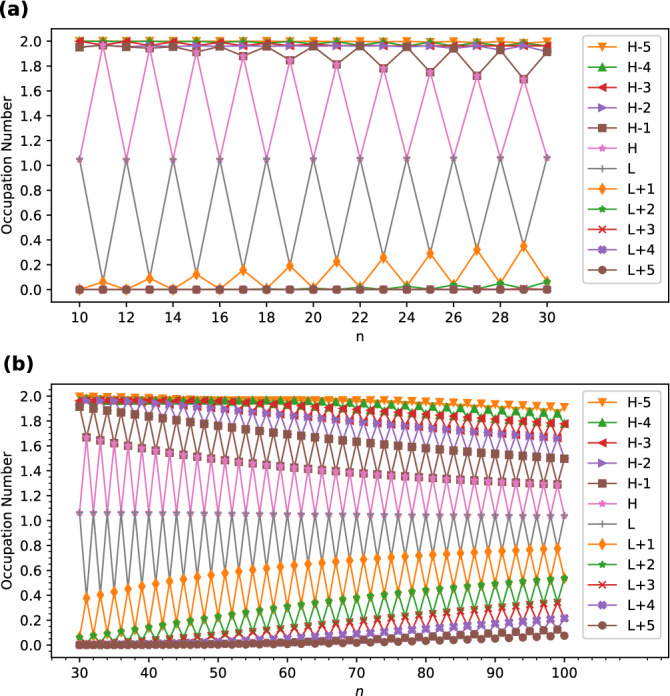
Active orbital occupation numbers (HOMO−5,...,HOMO−1, HOMO, LUMO, LUMO+1,...,and LUMO+5) of ground-state *l*-CC[*n*] with (**a**) *n* = 10–30 and (**b**) *n* = 30–100, calculated using spin-restricted TAO-LDA. For brevity, HOMO/LUMO is denoted as H/L.

As shown in Fig. 5, the active orbital occupation numbers of the smaller *l*-CC[*n*] (e.g., up to *n* = 20) reveal odd-even oscillation patterns, indicating that odd-numbered *l*-CC[*n*] possess nonradical nature (i.e., the occupation numbers of all orbitals are close to either 0 or 2), and even-numbered *l*-CC[*n*] possess pronounced diradical nature (i.e., the active orbitals are HOMO and LUMO). However, with the increase of the size of *l*-CC[*n*], the active orbital occupation numbers become closer to 1 and/or the number of active orbitals increases, suggesting an increasing polyradical nature of the larger *l*-CC[*n*].

**Figure 6:**
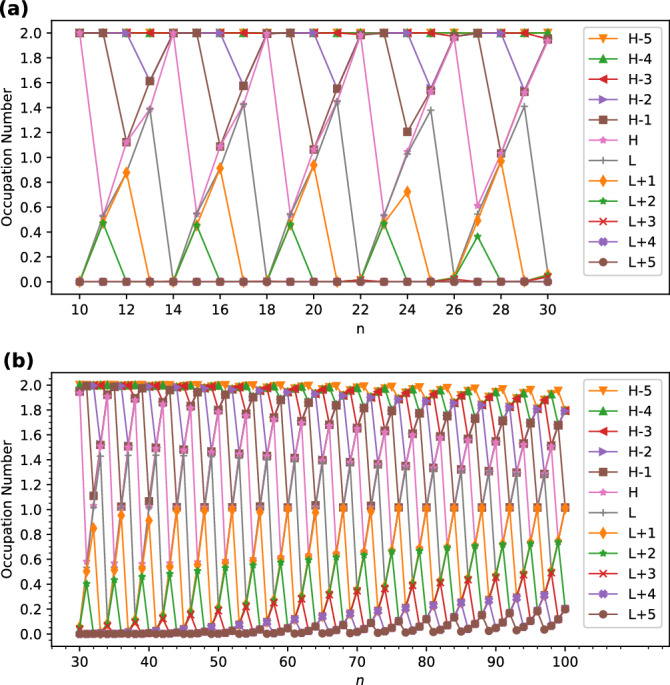
Active orbital occupation numbers (HOMO−5,...,HOMO−1, HOMO, LUMO, LUMO+1,...,and LUMO+5) of ground-state *c*-CC[*n*] with (**a**) *n* = 10–30 and (**b**) *n* = 30–100, calculated using spin-restricted TAO-LDA. For brevity, HOMO/LUMO is denoted as H/L.

In contrast to *l*-CC[*n*], the active orbital occupation numbers of *c*-CC[*n*] display very different patterns (see Fig. 6). In particular, the smaller *c*-CC[$4m+2$] (e.g., up to $4m+2$ = 46) possess nonradical nature (i.e., the occupation numbers of all orbitals are close to either 0 or 2), and hence are relatively more stable than *c*-CC[4*m*], *c*-CC[$4m+1$], and *c*-CC[$4m+3$], where *m* are positive integers. By contrast, *c*-CC[4*m*] (where *m* are positive integers) possess tetraradical nature (i.e., the active orbitals are HOMO−1, HOMO, LUMO, and LUMO+1). Nevertheless, with the increase of the size of *c*-CC[*n*], the active orbital occupation numbers become closer to 1 and/or the number of active orbitals increases, suggesting an increasing polyradical nature of the larger *c*-CC[*n*].

On the basis of the active orbital occupation numbers, the smaller odd-numbered *l*-CC[*n*] and the smaller *c*-CC[$4m+2$] (where *m* are positive integers) possess nonradical nature in their ground states, being consistent with the analyses of the other electronic properties (e.g., the larger $E_{\text {ST}}$ values, larger $E_{\text {SQ}}$ values, larger $E_{g}$ values, and smaller $S_{\text {vN}}$ values) of these relatively stable linear carbon chains and cyclic carbon chains, respectively. Accordingly, this study confirms the high stability of the smaller *c*-CC[$4m+2$]^24,25^, including the recently synthesized *c*-CC[18]^26^. On the other hand, *c*-CC[4*m*] (where *m* are positive integers) possess tetraradical nature in their ground states, also showing consistency with the analyses of the other electronic properties (e.g., the smaller $E_{\text {ST}}$ values, smaller $E_{\text {SQ}}$ values, smaller $E_{g}$ values, and larger $S_{\text {vN}}$ values) of these relatively unstable cyclic carbon chains.

### Real-space representation of active orbitals

**Figure 7:**
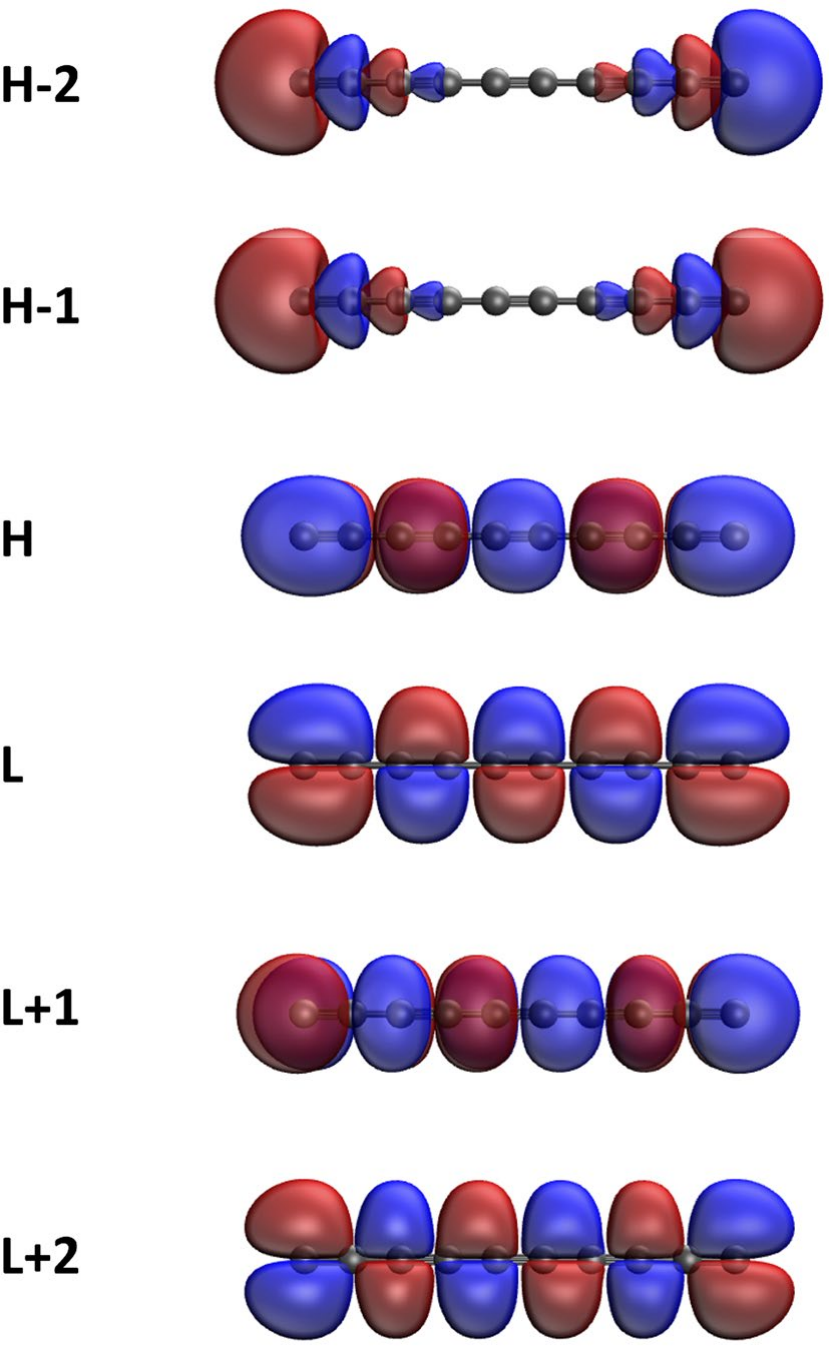
Real-space representation of HOMO−2 (1.952), HOMO−1 (1.951), HOMO (1.049), LUMO (1.049), LUMO+1 (0.000), and LUMO+2 (0.000) of ground-state *l*-CC[10], calculated using spin-restricted TAO-LDA, at isovalue = 0.02 e/Å$^3$. The orbital occupation numbers are given in parentheses. For brevity, HOMO/LUMO is denoted as H/L.

**Figure 8:**
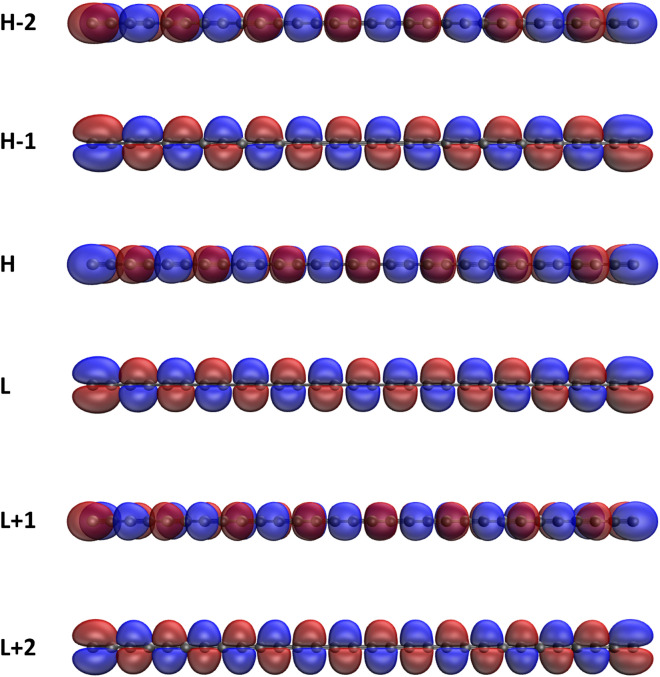
Real-space representation of HOMO−2 (1.914), HOMO−1 (1.914), HOMO (1.063), LUMO (1.063), LUMO+1 (0.064), and LUMO+2 (0.064) of ground-state *l*-CC[30], calculated using spin-restricted TAO-LDA, at isovalue = 0.02 e/Å$^3$. The orbital occupation numbers are given in parentheses. For brevity, HOMO/LUMO is denoted as H/L.

**Figure 9:**
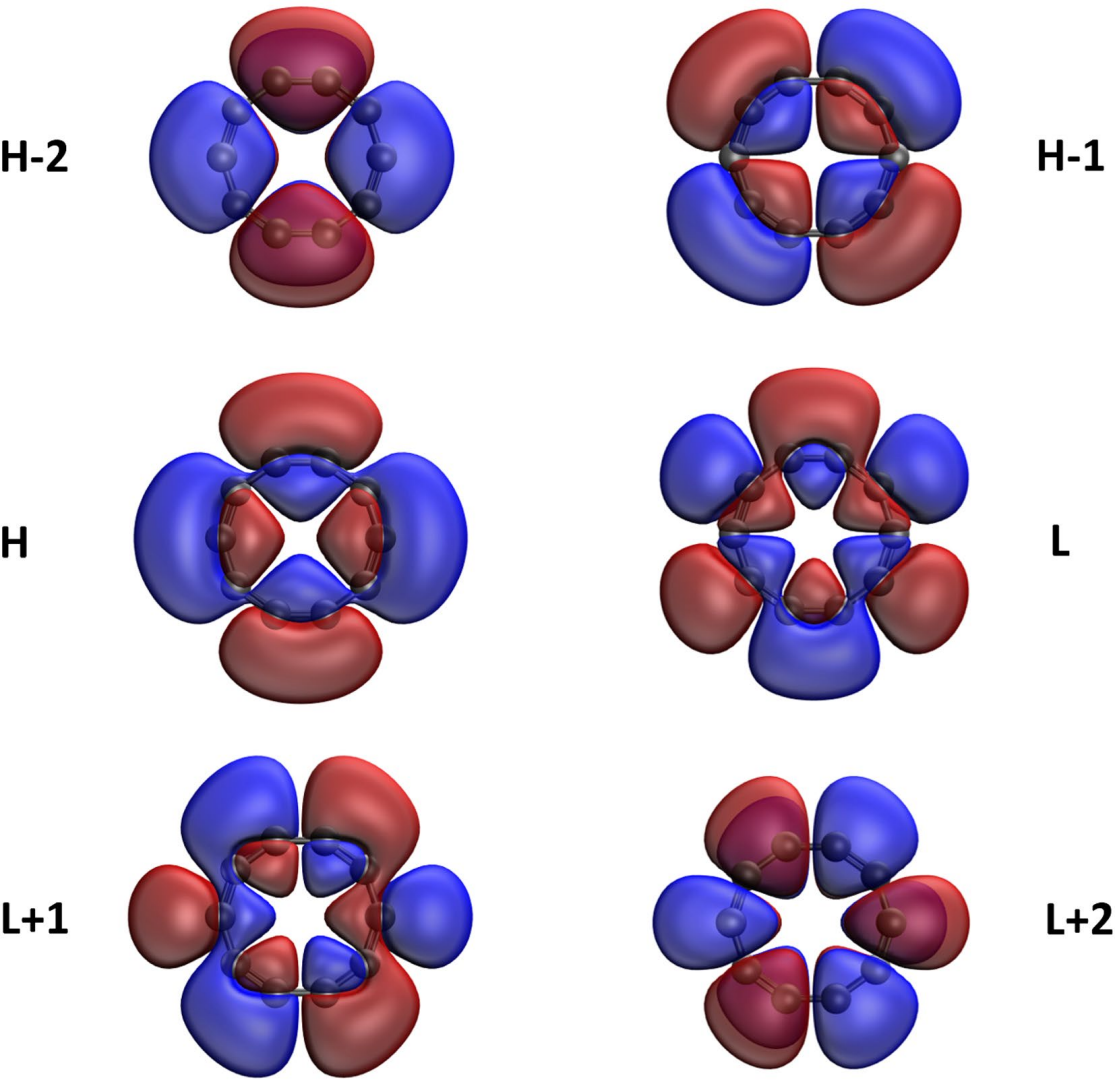
Real-space representation of HOMO−2 (2.000), HOMO−1 (2.000), HOMO (2.000), LUMO (0.000), LUMO+1 (0.000), and LUMO+2 (0.000) of ground-state *c*-CC[10], calculated using spin-restricted TAO-LDA, at isovalue = 0.02 e/Å$^3$. The orbital occupation numbers are given in parentheses. For brevity, HOMO/LUMO is denoted as H/L.

**Figure 10:**
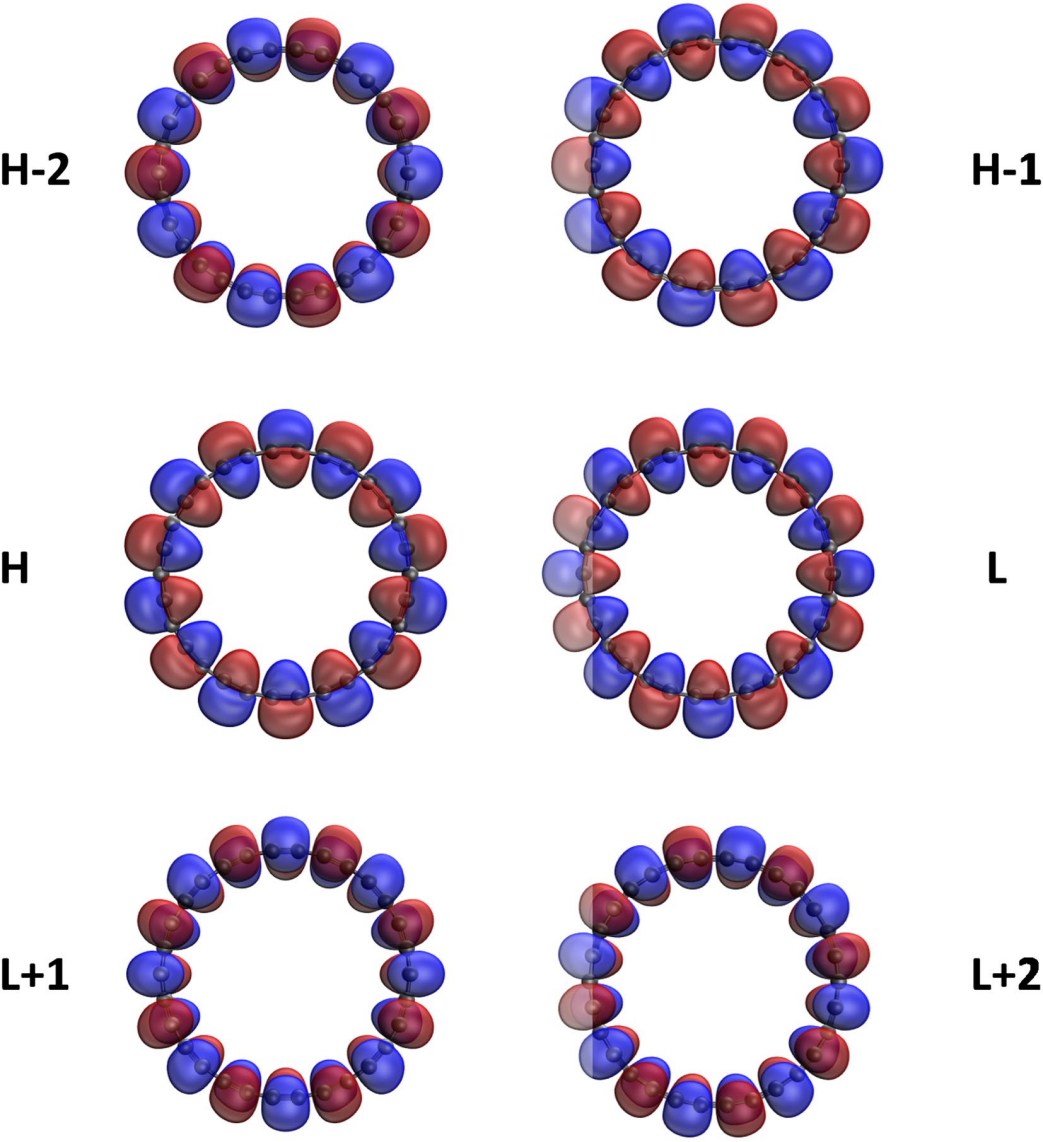
Real-space representation of HOMO−2 (1.950), HOMO−1 (1.949), HOMO (1.935), LUMO (0.066), LUMO+1 (0.057), and LUMO+2 (0.053) of ground-state *c*-CC[30], calculated using spin-restricted TAO-LDA, at isovalue = 0.02 e/Å$^3$. The orbital occupation numbers are given in parentheses. For brevity, HOMO/LUMO is denoted as H/L.

Here, we plot the real-space representation of the active orbitals, such as HOMO−2, HOMO−1, HOMO, LUMO, LUMO+1, and LUMO+2, for the ground states of some representative *l*-CC[*n*] (see Figs. 7 and 8) and *c*-CC[*n*] (see Figs. 9 and 10), obtained with spin-restricted TAO-LDA (also see Figures S1 to S6 in SI for more illustrative cases). The real-space representation analysis indicates that the active orbitals are delocalized over the entire length of *l*-CC[*n*] or the whole circumference of *c*-CC[*n*]. As the electrical conductivities of molecules consisting of many delocalized electrons are likely to be high^70^, it can be anticipated that *l*-CC[*n*]/*c*-CC[*n*] should be highly conductive due to the presence of delocalized electrons.

### Relative stability

We carry out spin-unrestricted TAO-LDA calculations to investigate the relative stability of *l*-CC[*n*] and *c*-CC[*n*] (i.e., the two isomers) in their ground states, which is examined by

7 $$\begin{aligned} E_{rel} = E_{\text {S}}({\textit{l-}CC}) - E_{\text {S}}({\textit{c-}CC}). \end{aligned}$$

Here, $E_{rel}$ is the relative energy of ground-state *l*-CC[*n*] with respect to ground-state *c*-CC[*n*], and $E_{\text {S}}({\textit{l-}CC})$ and $E_{\text {S}}({\textit{c-}CC})$ are the lowest singlet state (i.e., ground state) energies of *l*-CC[*n*] and *c*-CC[*n*], respectively.

**Figure 11:**
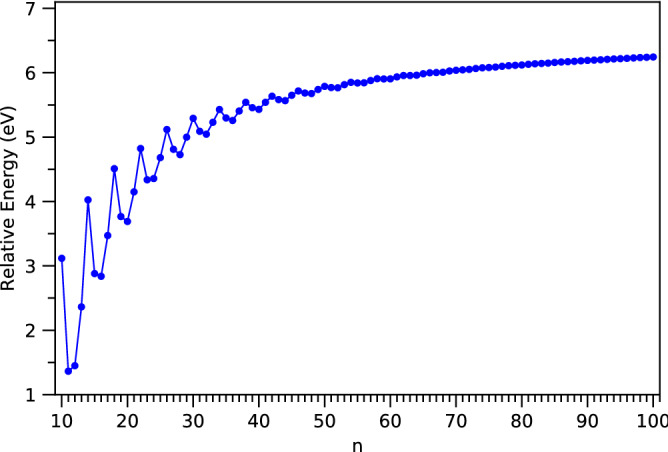
Relative energy of ground-state *l*-CC[*n*] with respect to ground-state *c*-CC[*n*] for *n* = 10–100, calculated using spin-unrestricted TAO-LDA.

The $E_{rel}$ value as a function of the number of carbon atoms is plotted in Fig. 11 (also see Table S5 in SI). As the system size increases, the $E_{rel}$ values are oscillatory only for smaller values of *n* (up to *n* = 59), and become monotonically increasing for larger *n*. For all the cases investigated, *c*-CC[*n*] are energetically more stable than *l*-CC[*n*], showing the significance of cyclic topology.

## Conclusions

In conclusion, we have employed TAO-DFT to investigate the electronic properties (e.g., the singlet-triplet energy gap, singlet-quintet energy gap, vertical ionization potential, vertical electron affinity, fundamental gap, symmetrized von Neumann entropy, active orbital occupation numbers, real-space representation of active orbitals, and relative stability) of *l*-CC[*n*]/*c*-CC[*n*] with *n* = 10–100 carbon atoms. For considerably large *n*, *l*-CC[*n*]/*c*-CC[*n*] are polyradicals in their ground states, playing an important role in determining their electronic properties. In view of their polyradical nature, it can be unreliable to study the properties of the larger *l*-CC[*n*]/*c*-CC[*n*] using KS-DFT with the traditional XC energy functionals, and it can be computationally intractable to study the properties of the larger *l*-CC[*n*]/*c*-CC[*n*] using accurate MR electronic structure methods. Consequently, it is well justified to study the electronic properties of *l*-CC[*n*]/*c*-CC[*n*] using TAO-DFT (i.e., a computationally efficient electronic structure method for nanosystems with radical nature) in this work.

For all the cases investigated, *l*-CC[*n*] and *c*-CC[*n*] are ground-state singlets, and *c*-CC[*n*] are energetically more stable than *l*-CC[*n*]. The electronic properties of *l*-CC[*n*] and *c*-CC[*n*] display peculiar oscillation patterns for smaller values of *n*, followed by monotonic changes for larger values of *n*. For the smaller carbon chains, odd-numbered *l*-CC[*n*] are more stable than the adjacent even-numbered ones, and *c*-CC[$4m+2$]/*c*-CC[4*m*] (where *m* are positive integers) are more/less stable than the adjacent odd-numbered ones. With the increase of *n*, *l*-CC[*n*] and *c*-CC[*n*] possess increasing polyradical nature in their ground states, with the active orbitals being delocalized over the entire length of *l*-CC[*n*] or the whole circumference of *c*-CC[*n*].

On the basis of our TAO-LDA results, the smaller *c*-CC[$4m+2$] (up to $4m+2$ = 22, where *m* are positive integers) possess nonradical nature and sizable singlet-triplet energy gaps (e.g., larger than 20 kcal/mol). In view of their high stability, it can be anticipated that these relatively stable cyclic carbon chains, such as *c*-CC[10], *c*-CC[14], *c*-CC[18], and *c*-CC[22], are likely to be synthesized in the near future. Note that among them, *c*-CC[18] have been recently synthesized^26^.

While the method adopted (i.e., TAO-LDA with the fictitious temperature $\theta$ = 7 mhartree^49^) is computationally efficient for the study of nanosystems with radical nature, a few limitations remain. First, owing to the use of LDA XC and $\theta$-dependent density functionals in TAO-DFT, TAO-LDA can yield the self-interaction error^41,49, 51^, and hence bond length alternation (BLA) can be suppressed^30^. Second, the present $\theta$ value is system-independent, and hence the static correlation associated with electronic systems can only be described approximately^53^. Note that the first issue can be greatly resolved by using the long-range corrected hybrid XC functionals^71,72^ in TAO-DFT^51,52^ for an improved description of nonlocal exchange effects, and the second issue can be greatly resolved by using the respective self-consistent scheme for determining the $\theta$ value^53^. Accordingly, we plan to work in this direction in the future.

On the other hand, to fully understand the impact of molecular geometries (e.g., BLA) on the electronic properties (e.g., the singlet-triplet energy gap, singlet-quintet energy gap, fundamental gap, and active orbital occupation numbers) of *l*-CC[*n*]/*c*-CC[*n*], it is essential to employ accurate multi-reference methods, such as the CASSCF (for static correlation) and possibly, CASPT2 (for both static correlation and dynamical correlation) methods, for geometry optimizations and single-point energy calculations on different spin states (e.g., the lowest singlet, triplet, and quintet states) of *l*-CC[*n*]/*c*-CC[*n*]. As this task can be computationally intractable, the electronic properties of *l*-CC[*n*]/*c*-CC[*n*] calculated using reasonably accurate and relatively inexpensive multi-reference methods are needed.

## Supplementary information

Supplementary Information 1.

## Data Availability

The data that support the findings of this study are available from the corresponding author upon reasonable request.
