# Supplementary material for: TAO-DFT investigation of electronic properties of linear and cyclic carbon chains
Source: Sci Rep. 2020 Aug 4;10:13133. doi: 10.1038/s41598-020-70023-z (PMC7403413; doi:10.1038/s41598-020-70023-z)
Supplement: Supplementary file 1 — Supplementary Information 1. [file 41598_2020_70023_MOESM1_ESM.pdf]

Supplementary Information to: TAO-DFT Investigation of Electronic Properties of Linear and Cyclic Carbon Chains

Sonai Seenithurai[1] and Jeng-Da Chai[1,2,*]

[1]*Department of Physics, National Taiwan University, Taipei 10617, Taiwan*

[2]*Center for Theoretical Physics and Center for Quantum Science and Engineering, National Taiwan University, Taipei 10617, Taiwan*

[*] Author to whom correspondence should be addressed. Electronic mail: jdchai@phys.ntu.edu.tw

LIST OF FIGURES

S1 Energy per atom for the lowest singlet/triplet/quintet state of *l*-CC[n] with n = 10–100, calculated using spin-unrestricted TAO-LDA. . . . .

S2 Energy per atom for the lowest singlet/triplet/quintet state of *c*-CC[n] with n = 10–100, calculated using spin-unrestricted TAO-LDA. . . . .

S3 Real-space representation of HOMO−2 (1.694), HOMO−1 (1.694), HOMO (1.054), LUMO (1.054), LUMO+1 (0.312), and LUMO+2 (0.312) of ground-state *l*-CC[60], calculated using spin-restricted TAO-LDA, at isovalue = 0.02 e/Å^3. The orbital occupation numbers are given in parentheses. For brevity, HOMO/LUMO is denoted as H/L. . . . .

S4 Real-space representation of HOMO−2 (1.581), HOMO−1 (1.581), HOMO (1.045), LUMO (1.045), LUMO+1 (0.442), and LUMO+2 (0.442) of ground-state *l*-CC[80], calculated using spin-restricted TAO-LDA, at isovalue = 0.02 e/Å^3. The orbital occupation numbers are given in parentheses. For brevity, HOMO/LUMO is denoted as H/L. . . . .

S5 Real-space representation of HOMO−2 (1.497), HOMO−1 (1.497), HOMO (1.040), LUMO (1.040), LUMO+1 (0.537), and LUMO+2 (0.537) of ground-state *l*-CC[100], calculated using spin-restricted TAO-LDA, at isovalue = 0.02 e/Å^3. The orbital occupation numbers are given in parentheses. For brevity, HOMO/LUMO is denoted as H/L. . . . .

S6 Real-space representation of HOMO−2 (1.941), HOMO−1 (1.017), HOMO (1.017), LUMO (1.004), LUMO+1 (1.003), and LUMO+2 (0.051) of ground-state *c*-CC[60], calculated using spin-restricted TAO-LDA, at isovalue = 0.02 e/Å^3. The orbital occupation numbers are given in parentheses. For brevity, HOMO/LUMO is denoted as H/L. . . . .

S7 Real-space representation of HOMO−2 (1.870), HOMO−1 (1.017), HOMO (1.016), LUMO (1.014), LUMO+1 (1.004), and LUMO+2 (0.121) of ground-state *c*-CC[80], calculated using spin-restricted TAO-LDA, at isovalue = 0.02 e/Å^3. The orbital occupation numbers are given in parentheses. For brevity, HOMO/LUMO is denoted as H/L. . . . .

S8 Real-space representation of HOMO−2 (1.791), HOMO−1 (1.016), HOMO (1.016), LUMO (1.011), LUMO+1 (1.011), and LUMO+2 (0.205) of ground-state c-CC[100], calculated using spin-restricted TAO-LDA, at isovalue = 0.02 e/Å^3. The orbital occupation numbers are given in parentheses. For brevity, HOMO/LUMO is denoted as H/L. . . . .

LIST OF TABLES

S1 Singlet-triplet energy gap E_{ST} (in kcal/mol) of l-CC[n]/c-CC[n], calculated using spin-unrestricted TAO-LDA. . . . .
S2 Singlet-quintet energy gap E_{SQ} (in kcal/mol) of l-CC[n]/c-CC[n], calculated using spin-unrestricted TAO-LDA. . . . .
S3 Vertical ionization potential IP$_v$ (in eV), vertical electron affinity EA$_v$ (in eV), fundamental gap E_g (in eV), and symmetrized von Neumann entropy S_{vN} of ground-state l-CC[n], calculated using spin-unrestricted TAO-LDA. . . . .
S4 Vertical ionization potential IP$_v$ (in eV), vertical electron affinity EA$_v$ (in eV), fundamental gap E_g (in eV), and symmetrized von Neumann entropy S_{vN} of ground-state c-CC[n], calculated using spin-unrestricted TAO-LDA. . . . .
S5 Relative energy E_{rel} (in eV) of ground-state l-CC[n] with respect to ground-state c-CC[n], calculated using spin-unrestricted TAO-LDA. . . . .

I. STABILITY OF THE SPIN-RESTRICTED AND SPIN-UNRESTRICTED TAO-LDA CALCULATIONS FOR THE LOWEST SINGLET STATES OF l-CC[n] AND c-CC[n]

Systems with radicaloid nature are highly challenging for KS-DFT with the conventional XC density functionals because of the symmetry-breaking effects that frequently occur in the corresponding spin-unrestricted calculations [1]. The symmetry constraint requires that the spin-restricted and spin-unrestricted calculations should be the same for an exact theory [2, 3]. Therefore, in order to check whether such unphysical symmetry-breaking effects occur in the spin-unrestricted TAO-LDA calculations, we also perform the spin-restricted TAO-LDA optimizations for the lowest singlet states of l-CC[n] and c-CC[n], and confirm that

this requirement can be fulfilled by TAO-LDA (i.e., the energies of spin-restricted and spin-unrestricted TAO-LDA calculations are the same within the numerical accuracy considered in this work) for all the l-CC[n] and c-CC[n] studied.

[1] Cohen, A. J., Mori-Sánchez, P. & Yang, W. Challenges for density functional theory. *Chem. Rev.* **112**, 289–320 (2012).

[2] Chai, J.-D. Density functional theory with fractional orbital occupations. *J. Chem. Phys.* **136**, 154104 (2012).

[3] Rivero, P., Jiménez-Hoyos, C. A. & Scuseria, G. E. Entanglement and polyradical character of polycyclic aromatic hydrocarbons predicted by projected Hartree-Fock theory. *J. Phys. Chem. B* **117**, 12750–12758 (2013).

FIGURES

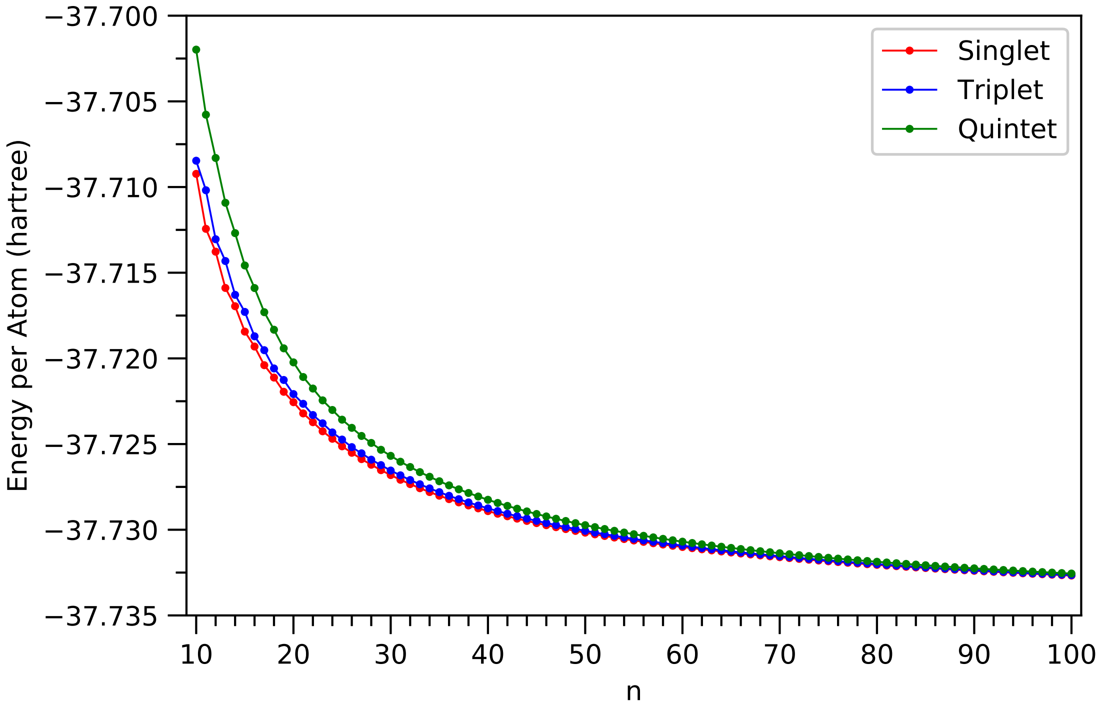

FIG. S1. Energy per atom for the lowest singlet/triplet/quintet state of l-CC[n] with $n = 10$–100, calculated using spin-unrestricted TAO-LDA.

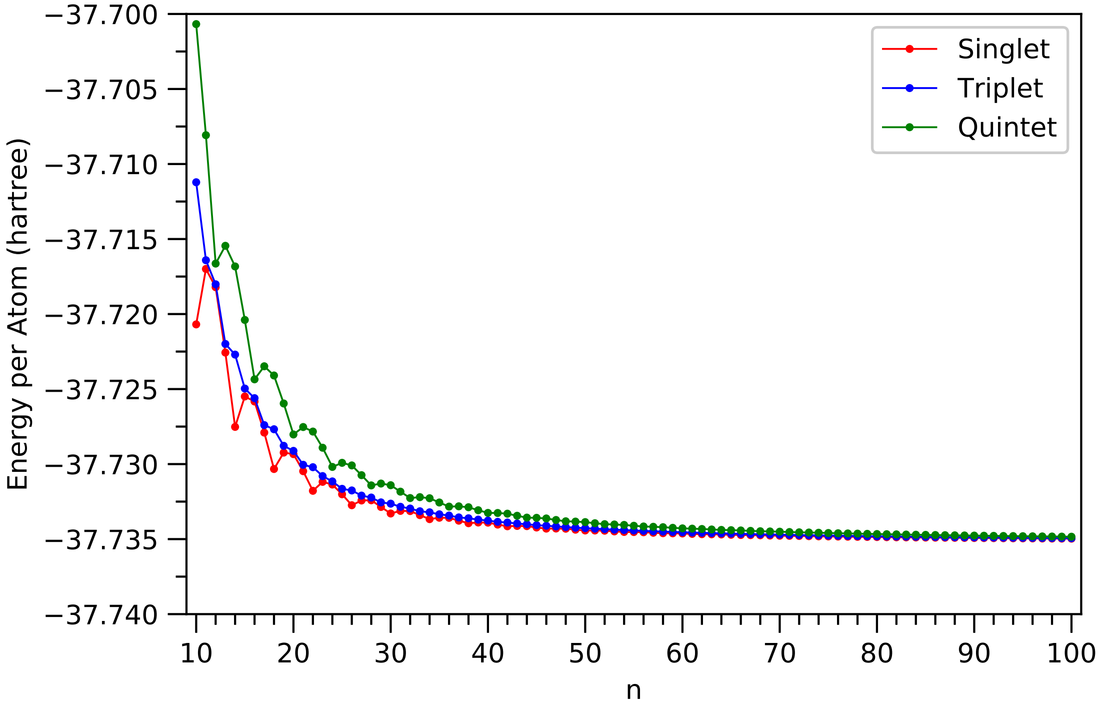

FIG. S2. Energy per atom for the lowest singlet/triplet/quintet state of c-CC[n] with $n = 10$–100, calculated using spin-unrestricted TAO-LDA.

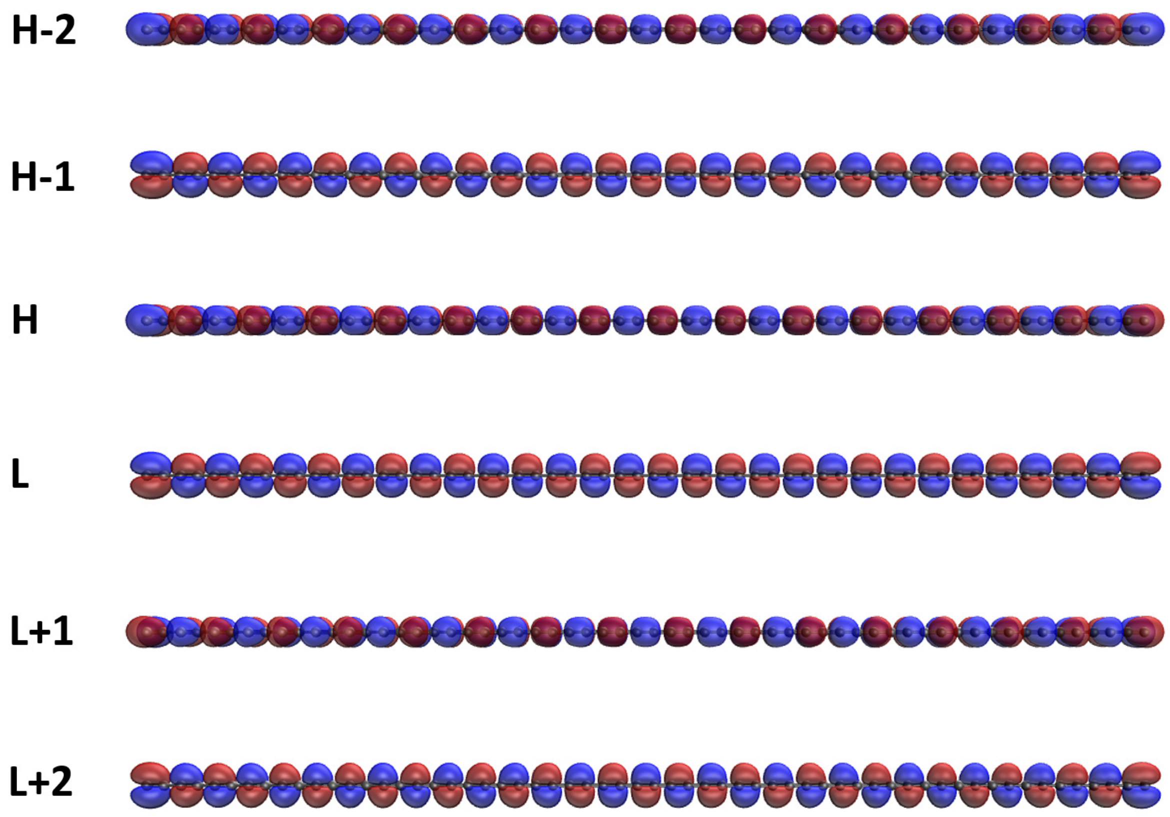

FIG. S3. Real-space representation of HOMO−2 (1.694), HOMO−1 (1.694), HOMO (1.054), LUMO (1.054), LUMO+1 (0.312), and LUMO+2 (0.312) of ground-state *l*-CC[60], calculated using spin-restricted TAO-LDA, at isovalue = 0.02 e/$\mathring{A}^3$. The orbital occupation numbers are given in parentheses. For brevity, HOMO/LUMO is denoted as H/L.

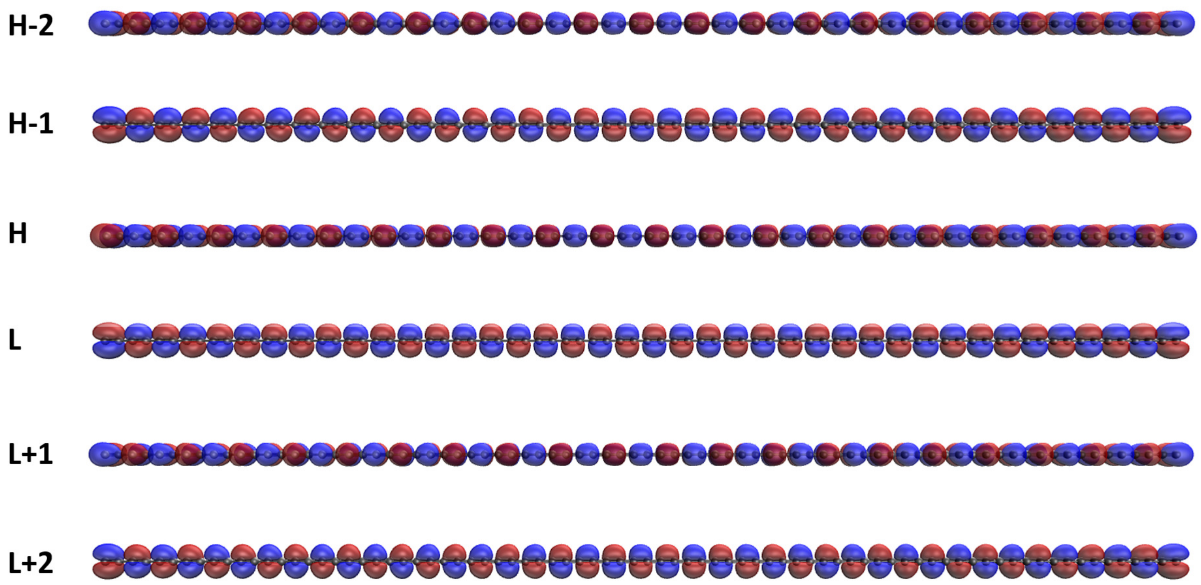

FIG. S4. Real-space representation of HOMO−2 (1.581), HOMO−1 (1.581), HOMO (1.045), LUMO (1.045), LUMO+1 (0.442), and LUMO+2 (0.442) of ground-state l-CC[80], calculated using spin-restricted TAO-LDA, at isovalue = 0.02 e/Å^3. The orbital occupation numbers are given in parentheses. For brevity, HOMO/LUMO is denoted as H/L.

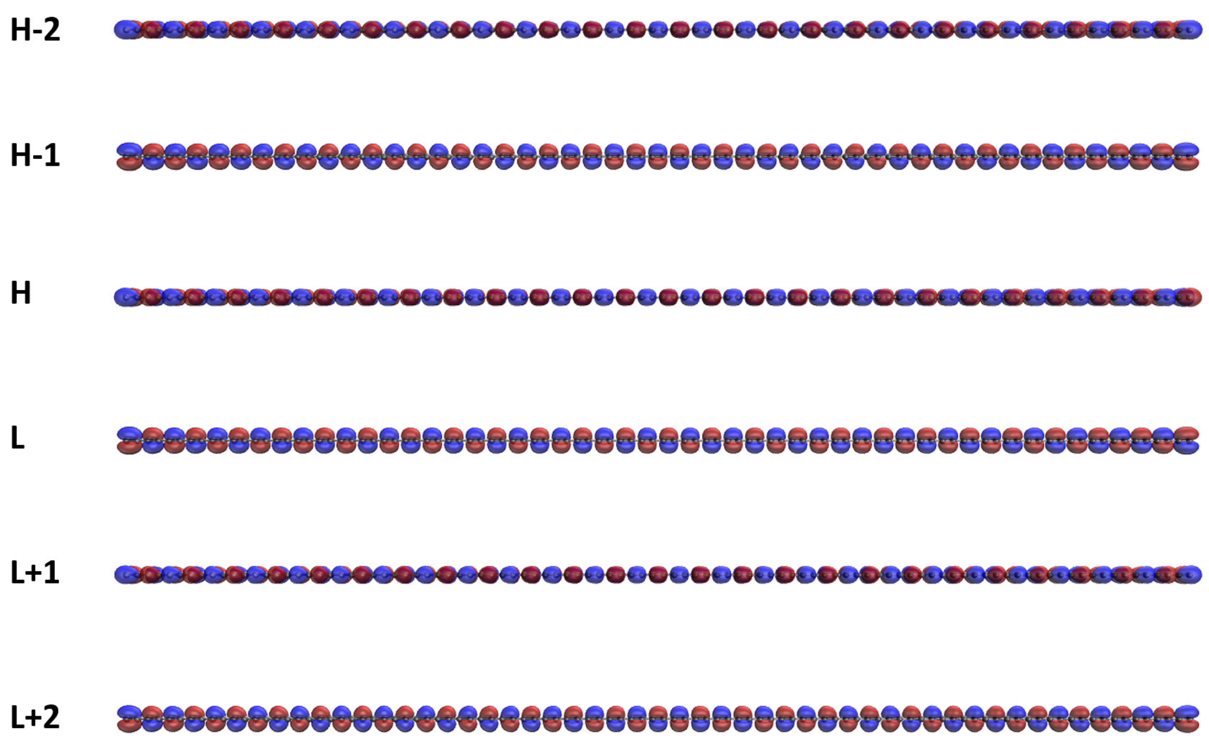

FIG. S5. Real-space representation of HOMO−2 (1.497), HOMO−1 (1.497), HOMO (1.040), LUMO (1.040), LUMO+1 (0.537), and LUMO+2 (0.537) of ground-state *l*-CC[100], calculated using spin-restricted TAO-LDA, at isovalue = 0.02 e/Å^3. The orbital occupation numbers are given in parentheses. For brevity, HOMO/LUMO is denoted as H/L.

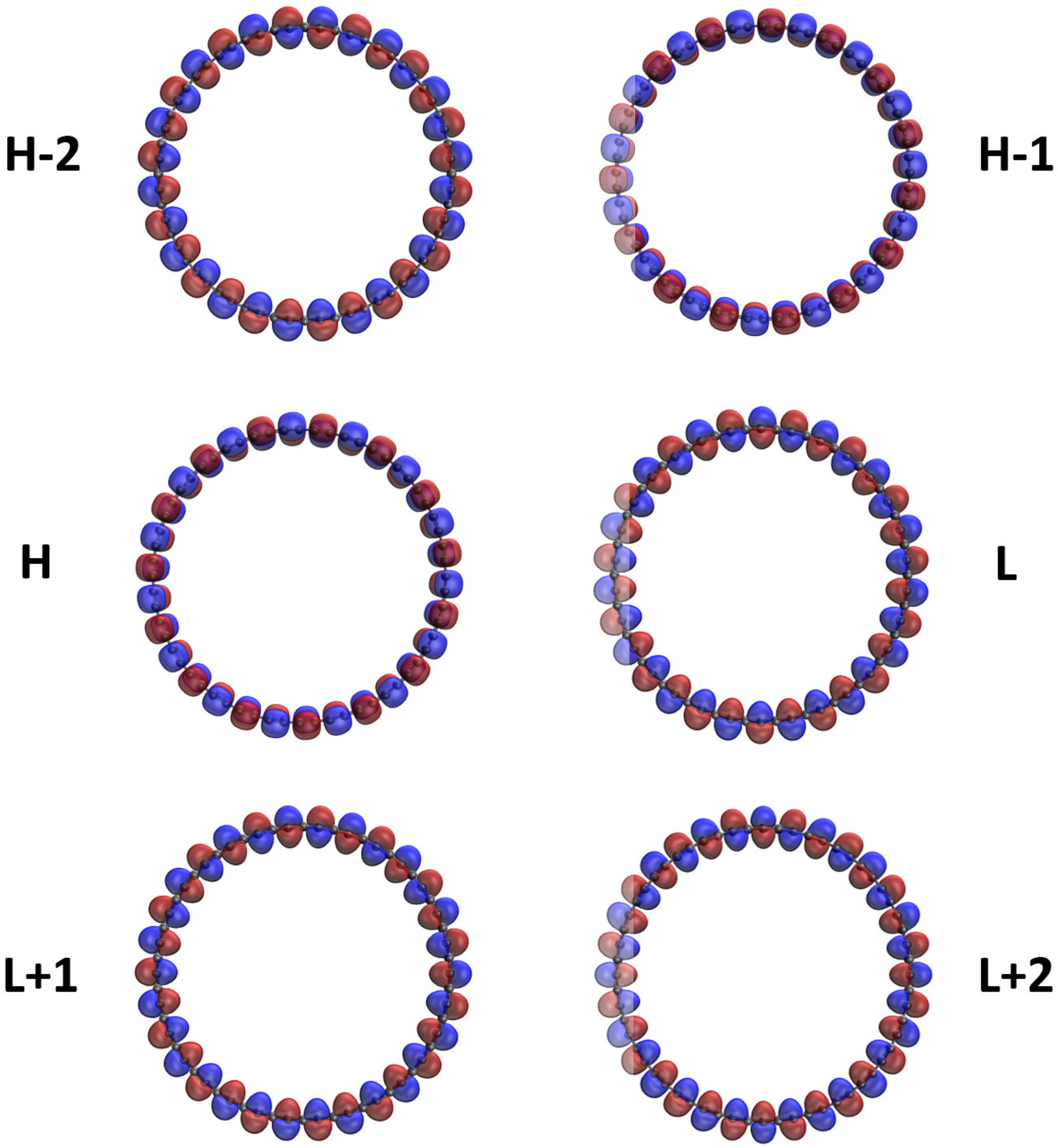

FIG. S6. Real-space representation of HOMO−2 (1.941), HOMO−1 (1.017), HOMO (1.017), LUMO (1.004), LUMO+1 (1.003), and LUMO+2 (0.051) of ground-state *c*-CC[60], calculated using spin-restricted TAO-LDA, at isovalue = 0.02 e/Å^3. The orbital occupation numbers are given in parentheses. For brevity, HOMO/LUMO is denoted as H/L.

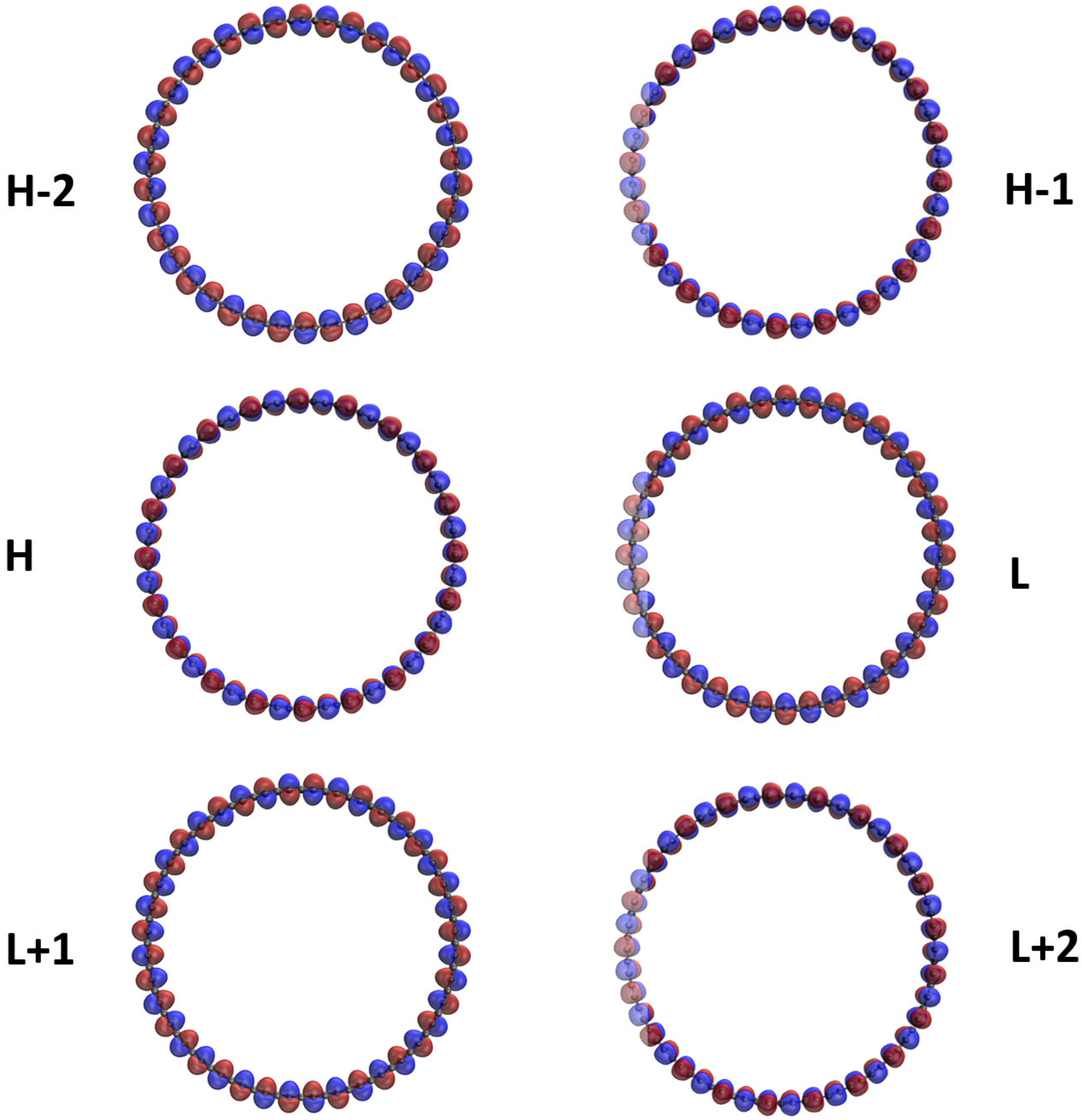

FIG. S7. Real-space representation of HOMO−2 (1.870), HOMO−1 (1.017), HOMO (1.016), LUMO (1.014), LUMO+1 (1.004), and LUMO+2 (0.121) of ground-state *c*-CC[80], calculated using spin-restricted TAO-LDA, at isovalue = 0.02 e/Å^3. The orbital occupation numbers are given in parentheses. For brevity, HOMO/LUMO is denoted as H/L.

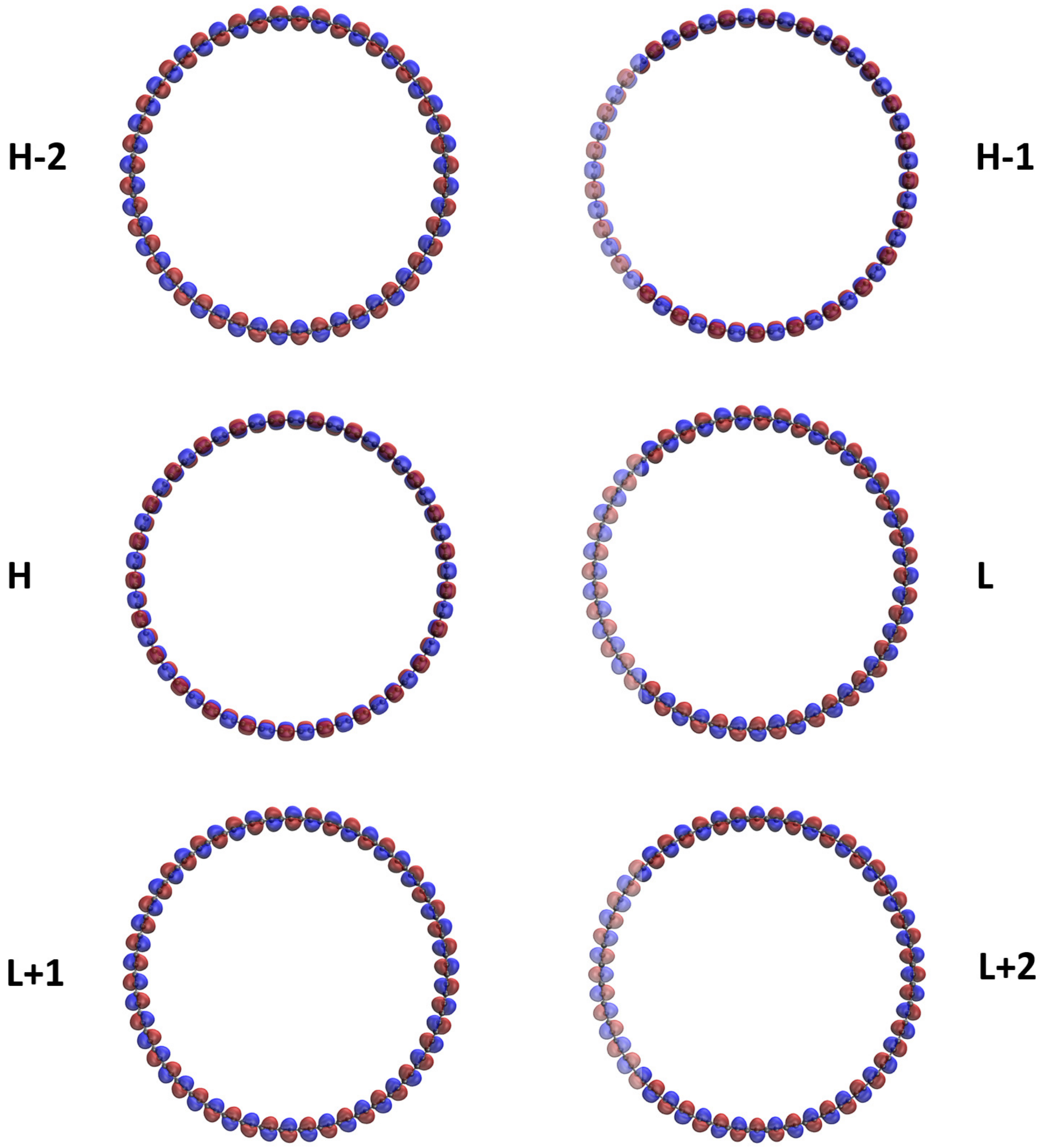

FIG. S8. Real-space representation of HOMO−2 (1.791), HOMO−1 (1.016), HOMO (1.016), LUMO (1.011), LUMO+1 (1.011), and LUMO+2 (0.205) of ground-state *c*-CC[100], calculated using spin-restricted TAO-LDA, at isovalue = 0.02 e/Å^3. The orbital occupation numbers are given in parentheses. For brevity, HOMO/LUMO is denoted as H/L.

TABLES

TABLE S1. Singlet-triplet energy gap E_{ST} (in kcal/mol) of l-CC[n]/c-CC[n], calculated using spin-unrestricted TAO-LDA.

n	E_{ST} (l-CC[n])	E_{ST} (c-CC[n])
10	4.81	59.44
11	15.54	4.02
12	5.47	1.46
13	12.82	4.68
14	5.84	42.33
15	10.83	4.97
16	6.00	2.24
17	9.35	5.31
18	6.01	29.93
19	8.23	5.41
20	5.93	2.70
21	7.37	5.61
22	5.78	21.72
23	6.70	5.54
24	5.59	3.13
25	6.16	5.69
26	5.38	16.07
27	5.72	5.51
28	5.17	3.18
29	5.36	5.56
30	4.96	12.33
31	5.05	5.30
32	4.75	3.39
33	4.78	5.33

34	4.56	9.70
35	4.54	5.02
36	4.37	3.43
37	4.33	5.03
38	4.20	7.84
39	4.14	4.71
40	4.04	3.45
41	3.97	4.70
42	3.88	6.50
43	3.82	4.39
44	3.74	3.42
45	3.68	4.38
46	3.61	5.53
47	3.54	4.10
48	3.48	3.36
49	3.42	4.07
50	3.37	4.81
51	3.31	3.82
52	3.26	3.33
53	3.20	3.79
54	3.16	4.26
55	3.10	3.57
56	3.06	3.27
57	3.01	3.53
58	2.97	3.83
59	2.92	3.35
60	2.88	3.05
61	2.84	3.30
62	2.80	3.53
63	2.76	3.15

64	2.73	2.93
65	2.69	3.10
66	2.65	3.18
67	2.62	2.97
68	2.59	2.77
69	2.55	2.92
70	2.52	2.99
71	2.49	2.81
72	2.46	2.77
73	2.43	2.76
74	2.40	2.84
75	2.37	2.66
76	2.35	2.59
77	2.32	2.61
78	2.29	2.63
79	2.27	2.53
80	2.24	2.46
81	2.22	2.48
82	2.19	2.49
83	2.17	2.39
84	2.14	2.35
85	2.12	2.39
86	2.10	2.36
87	2.08	2.28
88	2.06	2.26
89	2.04	2.27
90	2.02	2.28
91	2.00	2.20
92	1.98	2.16
93	1.96	2.14

94	1.94	2.15
95	1.92	2.09
96	1.90	2.08
97	1.88	2.06
98	1.87	2.06
99	1.85	2.02
100	1.83	1.99

TABLE S2. Singlet-quintet energy gap E_{SQ} (in kcal/mol) of l-CC[n]/c-CC[n], calculated using spin-unrestricted TAO-LDA.

n	E_{SQ} (l-CC[n])	E_{SQ} (c-CC[n])
10	45.50	125.58
11	45.97	61.54
12	41.18	11.86
13	40.55	58.05
14	37.48	93.95
15	36.34	48.00
16	34.28	14.83
17	32.99	47.14
18	31.53	70.39
19	30.26	39.04
20	29.16	16.43
21	27.98	38.77
22	27.10	54.50
23	26.04	32.82
24	25.31	17.67
25	24.37	32.88
26	23.74	43.29
27	22.91	28.59
28	22.35	17.41

29	21.62	28.40
30	21.12	35.48
31	20.48	24.90
32	20.01	17.41
33	19.45	24.84
34	19.02	29.71
35	18.52	22.31
36	18.13	16.86
37	17.68	22.20
38	17.32	25.38
39	16.91	20.01
40	16.58	16.28
41	16.21	19.91
42	15.90	22.08
43	15.56	18.31
44	15.28	15.60
45	14.97	18.16
46	14.70	19.51
47	14.42	16.75
48	14.17	14.89
49	13.91	16.58
50	13.67	17.49
51	13.43	15.49
52	13.21	14.16
53	12.99	15.30
54	12.78	15.86
55	12.58	14.40
56	12.38	13.49
57	12.19	14.20
58	12.01	14.53

59	11.82	13.50
60	11.65	12.71
61	11.48	13.28
62	11.32	13.43
63	11.16	12.69
64	11.00	12.14
65	10.85	12.48
66	10.71	12.53
67	10.56	11.93
68	10.43	11.47
69	10.29	11.71
70	10.16	11.70
71	10.03	11.27
72	9.91	11.00
73	9.78	11.08
74	9.66	11.00
75	9.55	10.66
76	9.44	10.41
77	9.32	10.47
78	9.22	10.39
79	9.11	10.15
80	9.01	9.93
81	8.91	9.92
82	8.81	9.86
83	8.71	9.63
84	8.62	9.46
85	8.53	9.47
86	8.44	9.39
87	8.35	9.19
88	8.26	9.07

89	8.18	9.02
90	8.09	8.95
91	8.01	8.79
92	7.93	8.68
93	7.86	8.62
94	7.78	8.56
95	7.70	8.44
96	7.63	8.32
97	7.56	8.26
98	7.49	8.21
99	7.42	8.10
100	7.35	8.00

TABLE S3. Vertical ionization potential IP_v (in eV), vertical electron affinity EA_v (in eV), fundamental gap E_g (in eV), and symmetrized von Neumann entropy S_{vN} of ground-state l-CC[n], calculated using spin-unrestricted TAO-LDA.

n	IP_v	EA_v	E_g	S_{vN}
10	8.74	3.82	4.92	3.24
11	8.66	3.60	5.06	1.20
12	8.38	3.91	4.47	3.25
13	8.30	3.77	4.53	1.61
14	8.10	3.98	4.11	3.29
15	8.02	3.90	4.12	2.01
16	7.86	4.05	3.81	3.35
17	7.79	4.00	3.79	2.39
18	7.67	4.11	3.56	3.45
19	7.60	4.09	3.51	2.75
20	7.50	4.17	3.34	3.58
21	7.44	4.15	3.28	3.08
22	7.36	4.21	3.14	3.73

23	7.30	4.21	3.08	3.40
24	7.23	4.26	2.97	3.90
25	7.17	4.26	2.91	3.69
26	7.12	4.30	2.82	4.09
27	7.07	4.31	2.76	3.97
28	7.02	4.34	2.68	4.29
29	6.97	4.34	2.63	4.24
30	6.93	4.37	2.56	4.50
31	6.89	4.38	2.51	4.50
32	6.85	4.40	2.45	4.72
33	6.81	4.41	2.40	4.76
34	6.78	4.43	2.35	4.94
35	6.74	4.44	2.30	5.01
36	6.71	4.45	2.26	5.17
37	6.68	4.46	2.21	5.26
38	6.65	4.47	2.17	5.40
39	6.62	4.48	2.13	5.50
40	6.59	4.50	2.09	5.64
41	6.56	4.51	2.06	5.75
42	6.54	4.52	2.02	5.88
43	6.51	4.52	1.99	5.99
44	6.49	4.53	1.96	6.11
45	6.47	4.54	1.92	6.23
46	6.45	4.55	1.89	6.35
47	6.42	4.56	1.86	6.47
48	6.40	4.57	1.84	6.59
49	6.38	4.58	1.81	6.71
50	6.37	4.58	1.78	6.83
51	6.35	4.59	1.76	6.95
52	6.33	4.60	1.73	7.07

53	6.31	4.60	1.71	7.19
54	6.30	4.61	1.68	7.31
55	6.28	4.62	1.66	7.43
56	6.26	4.62	1.64	7.55
57	6.25	4.63	1.62	7.68
58	6.23	4.64	1.60	7.79
59	6.22	4.64	1.58	7.92
60	6.20	4.65	1.56	8.04
61	6.19	4.65	1.54	8.16
62	6.18	4.66	1.52	8.28
63	6.16	4.66	1.50	8.40
64	6.15	4.67	1.48	8.52
65	6.14	4.67	1.47	8.64
66	6.13	4.68	1.45	8.76
67	6.12	4.68	1.43	8.88
68	6.11	4.69	1.42	9.00
69	6.09	4.69	1.40	9.12
70	6.08	4.70	1.39	9.24
71	6.07	4.70	1.37	9.36
72	6.06	4.71	1.36	9.48
73	6.05	4.71	1.34	9.61
74	6.04	4.71	1.33	9.73
75	6.03	4.72	1.32	9.85
76	6.02	4.72	1.30	9.97
77	6.01	4.72	1.29	10.09
78	6.01	4.73	1.28	10.21
79	6.00	4.73	1.26	10.33
80	5.99	4.74	1.25	10.45
81	5.98	4.74	1.24	10.57
82	5.97	4.74	1.23	10.69

83	5.96	4.75	1.22	10.81
84	5.96	4.75	1.21	10.93
85	5.95	4.75	1.20	11.05
86	5.94	4.76	1.19	11.18
87	5.93	4.76	1.17	11.30
88	5.93	4.76	1.16	11.42
89	5.92	4.76	1.15	11.54
90	5.91	4.77	1.14	11.66
91	5.90	4.77	1.13	11.78
92	5.90	4.77	1.13	11.90
93	5.89	4.78	1.12	12.02
94	5.88	4.78	1.11	12.14
95	5.88	4.78	1.10	12.26
96	5.87	4.78	1.09	12.38
97	5.87	4.79	1.08	12.51
98	5.86	4.79	1.07	12.63
99	5.85	4.79	1.06	12.75
100	5.85	4.79	1.05	12.87

TABLE S4. Vertical ionization potential IP_v (in eV), vertical electron affinity EA_v (in eV), fundamental gap E_g (in eV), and symmetrized von Neumann entropy S_{vN} of ground-state c-CC[n], calculated using spin-unrestricted TAO-LDA.

n	IP_v	EA_v	E_g	S_{vN}
10	8.75	0.55	8.19	0.01
11	6.86	1.40	5.46	4.50
12	7.34	2.23	5.11	5.49
13	7.80	2.76	5.04	4.43
14	8.00	1.53	6.47	0.08
15	6.77	2.13	4.64	4.49
16	7.07	2.71	4.35	5.52

17	7.39	3.05	4.33	4.47
18	7.50	2.24	5.26	0.30
19	6.68	2.63	4.05	4.50
20	6.86	3.05	3.80	5.54
21	7.09	3.27	3.81	4.50
22	7.14	2.74	4.41	0.72
23	6.59	2.99	3.60	4.52
24	6.71	3.31	3.40	5.45
25	6.87	3.45	3.42	4.52
26	6.89	3.10	3.79	1.31
27	6.51	3.27	3.24	4.55
28	6.56	3.50	3.07	5.61
29	6.69	3.59	3.10	4.61
30	6.68	3.36	3.33	1.98
31	6.43	3.47	2.95	4.70
32	6.46	3.65	2.81	5.66
33	6.54	3.71	2.84	4.76
34	6.53	3.56	2.97	2.70
35	6.35	3.64	2.71	4.91
36	6.36	3.77	2.59	5.83
37	6.42	3.81	2.61	4.98
38	6.40	3.71	2.69	3.43
39	6.28	3.77	2.51	5.19
40	6.27	3.87	2.40	6.00
41	6.32	3.89	2.43	5.27
42	6.30	3.83	2.46	4.14
43	6.21	3.88	2.33	5.52
44	6.21	3.95	2.26	6.25
45	6.23	3.97	2.26	5.63
46	6.21	3.94	2.28	4.83

47	6.15	3.97	2.18	5.91
48	6.14	4.03	2.12	6.53
49	6.16	4.04	2.12	6.03
50	6.14	4.02	2.12	5.48
51	6.10	4.05	2.05	6.33
52	6.09	4.09	2.00	6.85
53	6.10	4.10	2.00	6.47
54	6.08	4.09	1.99	6.10
55	6.05	4.11	1.93	6.77
56	6.04	4.14	1.90	7.20
57	6.04	4.16	1.89	6.93
58	6.03	4.15	1.88	6.69
59	6.00	4.17	1.83	7.22
60	5.99	4.20	1.80	7.59
61	6.00	4.21	1.79	7.40
62	5.98	4.21	1.77	7.26
63	5.96	4.22	1.74	7.69
64	5.95	4.24	1.71	8.00
65	5.95	4.25	1.70	7.87
66	5.94	4.25	1.69	7.81
67	5.92	4.27	1.66	8.16
68	5.92	4.28	1.63	8.42
69	5.91	4.29	1.62	8.36
70	5.90	4.30	1.61	8.34
71	5.89	4.31	1.58	8.64
72	5.88	4.32	1.56	8.86
73	5.88	4.33	1.55	8.84
74	5.87	4.33	1.54	8.87
75	5.86	4.34	1.52	9.12
76	5.85	4.35	1.50	9.31

77	5.85	4.36	1.49	9.33
78	5.84	4.37	1.47	9.38
79	5.83	4.38	1.45	9.60
80	5.82	4.39	1.44	9.77
81	5.82	4.39	1.43	9.81
82	5.81	4.40	1.41	9.88
83	5.80	4.41	1.40	10.08
84	5.80	4.41	1.38	10.24
85	5.79	4.42	1.37	10.30
86	5.79	4.43	1.36	10.38
87	5.78	4.43	1.35	10.56
88	5.77	4.44	1.33	10.71
89	5.77	4.45	1.32	10.79
90	5.76	4.45	1.31	10.88
91	5.76	4.46	1.30	11.04
92	5.75	4.47	1.29	11.18
93	5.75	4.47	1.28	11.27
94	5.74	4.48	1.27	11.37
95	5.74	4.48	1.25	11.52
96	5.73	4.49	1.24	11.66
97	5.73	4.49	1.23	11.76
98	5.72	4.50	1.22	11.86
99	5.72	4.50	1.21	12.01
100	5.71	4.51	1.20	12.14

TABLE S5. Relative energy E_{rel} (in eV) of ground-state l-CC[n] with respect to ground-state c-CC[n], calculated using spin-unrestricted TAO-LDA.

n	E_{rel}
10	3.12
11	1.36

12	1.45
13	2.36
14	4.02
15	2.88
16	2.84
17	3.47
18	4.51
19	3.77
20	3.69
21	4.15
22	4.82
23	4.34
24	4.36
25	4.68
26	5.12
27	4.81
28	4.73
29	5.00
30	5.29
31	5.09
32	5.05
33	5.23
34	5.43
35	5.30
36	5.26
37	5.41
38	5.54
39	5.46
40	5.43
41	5.54

42	5.64
43	5.58
44	5.57
45	5.65
46	5.72
47	5.68
48	5.68
49	5.74
50	5.79
51	5.77
52	5.77
53	5.81
54	5.85
55	5.84
56	5.84
57	5.88
58	5.91
59	5.90
60	5.90
61	5.94
62	5.96
63	5.96
64	5.96
65	5.98
66	6.00
67	6.00
68	6.01
69	6.03
70	6.04
71	6.04

72	6.05
73	6.07
74	6.08
75	6.08
76	6.09
77	6.10
78	6.11
79	6.11
80	6.12
81	6.13
82	6.14
83	6.14
84	6.15
85	6.16
86	6.17
87	6.17
88	6.18
89	6.18
90	6.19
91	6.20
92	6.20
93	6.21
94	6.21
95	6.22
96	6.22
97	6.23
98	6.24
99	6.24
100	6.24
